# Food Allergy Knowledge, Attitudes, and Practices Among Restaurant's Staff in Bangladesh: A Structural Modeling Approach

**DOI:** 10.1002/fsn3.70754

**Published:** 2025-08-03

**Authors:** Nitai Roy, Sultan Mahmud Imran, Sourav Chandra Debnath, Abdullah Al Adib, Samia Sultana, Sumana Mahmud, Rejwana Rashid, Ekhtear Hossain, Farhadul Islam, Kamal Krishna Biswas

**Affiliations:** ^1^ Department of Biochemistry and Molecular Biology Patuakhali Science and Technology University Patuakhali Bangladesh; ^2^ Faculty of Nutrition and Food Science Patuakhali Science and Technology University Patuakhali Bangladesh; ^3^ Department of Biological Sciences and Chemistry Southern University and A&M College Baton Rouge Louisiana USA; ^4^ Department of Biochemistry and Molecular Biology University of Rajshahi Rajshahi Bangladesh

**Keywords:** attitudes, food allergy, knowledge, practices, restaurant staff, structural equation modeling

## Abstract

Food‐related allergic reactions are increasingly prevalent in restaurant settings, often leading to severe and life‐threatening incidents. This study aimed to investigate the relationships among knowledge, attitudes, and management practices related to food allergies in restaurant staff in Bangladesh. A cross‐sectional study conducted between January and May 2024 assessed food allergy knowledge, attitudes, and practices among 712 restaurant staff across eight divisional areas in Bangladesh, using structured face‐to‐face interviews. Descriptive and structural equation modeling (SEM) were applied to validate the hypothesized model and assess latent constructs. The proposed model met the criteria for goodness of fit indices, indicating an acceptable fit to the data. Overall, restaurant staff members demonstrated a relatively higher level of knowledge; however, a significant amount of uncertainty was observed in their attitudes. Despite implementing effective measures to prevent cross‐contamination and safeguard against allergies, their management practices regarding customer communication and remaking meals following mistakes were insufficient for ensuring customer safety. Notably, knowledge had no significant impact on management practices, whereas attitudes toward food allergies had a strong and positive influence on how practices were implemented. There was also a positive association between knowledge and attitude, suggesting that a decline in knowledge is likely to correspond with a decrease in attitude scores. Two of our hypotheses (H2 and H3) were supported by the findings of this study. In conclusion, while restaurant staff exhibited a solid foundational knowledge and implemented effective practices to manage food allergies, gaps in attitudes and certain management practices highlight the need for enhanced training to ensure comprehensive allergy safety for all customers.

## Graphical abstract

Restaurant staff in Bangladesh had good foundational knowledge of food allergies, but attitudes showed notable uncertainty. Attitudes, but not knowledge, significantly influenced food allergy management practices, especially customer communication and corrective actions. The study supports enhancing staff training focused on attitude improvement to ensure comprehensive food allergy safety in restaurants.

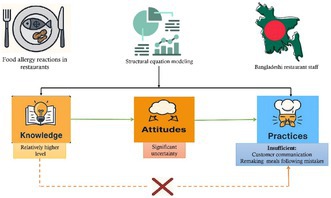

## 1 Introduction

Food allergy represents a serious and growing health concern, where even minimal exposure can trigger severe reactions. This underscores the importance of comprehensive knowledge, mindful attitudes, and safe practices to ensure a safe and enjoyable dining experience for all. Food allergies impact about 1%–10% of the global population, increasingly affecting infants, children, and adults worldwide (Gupta et al. 2023; Warren et al. 2020). In recent years, there has been a notable rise in the prevalence of food allergies within developing nations, a trend that contrasts sharply with earlier research (Leung et al. 2018).

Food allergy is an immune reaction to proteins found in food, which can be categorized as either immunoglobulin (Ig)E‐mediated or non–(Ig)E‐mediated. This condition often presents a diverse array of symptoms impacting the skin, gastrointestinal tract, and respiratory tract, with manifestations that can vary from mild irritations to severe, life‐threatening reactions (Lopez 2023; Moore et al. 2017). The landscape of food allergies is complex, with more than 160 foods identified as potential triggers for allergic reactions. However, the Food Allergen Labeling and Consumer Protection Act (FALCPA) brings attention to eight prevalent allergens: eggs, milk, fish (including bass and cod), shellfish (such as crab and shrimp), peanuts, wheat, tree nuts (like almonds and walnuts), and soybeans. These account for 90% of all food allergy reactions within the general population, underscoring their significance in food safety and consumer protection (FALCPA 2004; USDA 2024).

In Bangladesh, the food industry adheres to the Packaged Food Labelling Act of 2017, which mandates that packaged or processed food labels clearly indicate any ingredients that may trigger allergic or intolerant reactions. This regulation identifies 12 allergenic foods: gluten‐containing foods (such as barley, rye, wheat, and wheat grain), eggs, milk (including lactose), mustard, soybeans, peanuts, fish, sesame seeds, celery, pine nuts (such as almonds, hazelnuts, and walnuts), crustacean shellfish, and food products containing sulfites (10 mg per kg or more). Ensuring these allergens are included on the product label is crucial for consumer safety (Bangladesh Food Safety Authority 2017).

A study in Bangladesh found that brinjal, beef, and hilsa fish are among the most common foods causing allergic reactions (Ali et al. 2020). Since food allergies are incurable, prevention and management rely on avoiding known food allergens (FDA 2024). However, avoiding allergen‐containing foods can be difficult, especially in Bangladesh, where many people eat out or in restaurants, which is a growing concern (Ahmed 2023). Restaurants are a major site of allergic reactions, with the Centers for Disease Control and Prevention (CDC) reporting that one in three people with food allergies experience a reaction while dining out (CDC 2024). Miscommunication and lack of knowledge among restaurant staff frequently contribute to accidental exposures (Carter et al. 2020). A systematic review indicates that 21%–31% of allergic reactions occur in restaurants, often due to cross‐contamination during food preparation (Versluis et al. 2015). Improving restaurant practices and increasing staff knowledge about food allergies can help lower the risk of allergic reactions in restaurant settings (CDC 2024). However, studies reveal that restaurant workers often lack adequate knowledge to safely prepare meals for allergic customers (Ahuja and Sicherer 2007). Several studies have examined the knowledge, attitudes, and practices (KAP) of restaurant staff, focusing on their training and management of food allergens, highlighting the positive impact of training programs (Dupuis et al. 2016; Elsahoryi et al. 2021; Lee and Sozen 2016; Radke et al. 2016). Trained staff are better able to comprehend food allergies and effectively communicate with customers about potential concerns, contributing to a safer dining environment (Bailey et al. 2014; CDC 2024; Lee and Sozen 2016). Having good knowledge of food allergens, along with the correct attitude and proper practices by restaurant staff, can help reduce allergic reactions among individuals with food allergies.

While food safety KAP studies have been conducted in Bangladesh (Hashanuzzaman et al. 2020; Islam et al. 2022; Jubayer et al. 2020), there is a lack of research specifically addressing food allergy KAP among restaurant staff. To our knowledge, no studies have addressed this issue in Bangladesh. Additionally, the application of structural equation modeling (SEM) in food allergen research is limited (Soon 2018b, 2019), with only one study utilizing SEM to analyze food allergy KAP among restaurant workers (Agwa 2023). Although SEM has been applied in food safety KAP studies in Bangladesh (Hasan et al. 2022; Sarma et al. 2022), its use in food allergy research is unexplored. To address these gaps, this study examines the knowledge of food allergies, attitudes toward food allergens, and food allergy management practices among restaurant staff members in Bangladesh using the SEM method. In this study, the author introduces the following hypotheses, as suggested by several studies (Baser et al. 2017; Lim et al. 2016; Soon 2018b, 2019). The hypotheses are presented below:

*Knowledge of food allergies does not have a direct impact on food allergy management practices*.

*Attitude toward food allergies have a direct impact on food allergy management practices*.

*There is a correlation between knowledge and attitudes regarding food allergies*.

## 2 Materials and Methods

### 2.1 Study Purpose and Model

**FIGURE 1:**
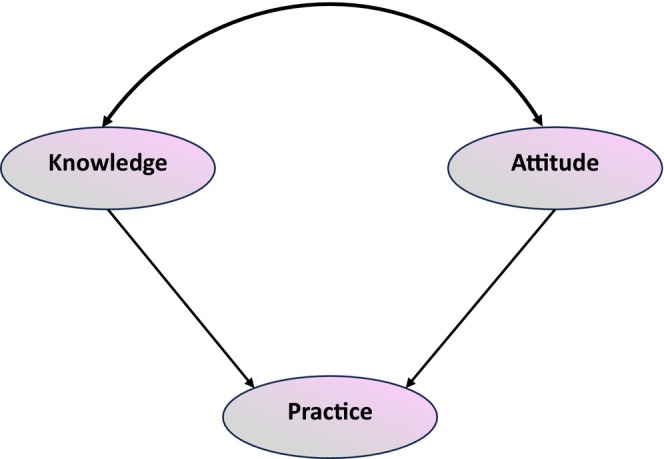
Model development of food allergen knowledge, attitude, and practices.

In the context of restaurant management, it is essential to emphasize the KAP related to food allergies. The primary causes of allergic reactions in commercial restaurants include cross‐contamination, hidden food allergies, and insufficient awareness among restaurant workers (Lee and Sozen 2016). The objective of this study was to evaluate the KAP of restaurant workers in Bangladesh with respect to food allergies and to investigate the interrelations between these factors through SEM. The objective of this research was to identify gaps in the management of food allergies, emphasize the potential for cross‐contamination, and offer suggestions for enhancing the safety protocols of restaurants. SEM is particularly well‐suited for this study because it enables the simultaneous analysis of numerous interrelated dependent and independent variables, thereby facilitating a comprehensive comprehension of the dynamic interactions between KAP factors. The conceptual model depicted in Figure 1 serves as the basis for analysis in this study.

### 2.2 Sampling and Data Collection

A cross‐sectional study was conducted from January 2024 to May 2024 to evaluate the KAP of staff at restaurants in Bangladesh with respect to food allergies. The study included all eight divisional areas: Dhaka, Rangpur, Rajshahi, Barisal, Khulna, Sylhet, Mymensingh, and Chittagong. Potential respondents were provided with an information sheet that explained the study's objectives, significance, and procedures before their participation. All participants were granted informed consent before participating in the survey. Face‐to‐face interviews were conducted by a team of trained interviewers, who employed a structured questionnaire to collect data. This approach was selected to enable a thorough comprehension of the participants' practical experiences in addressing food allergies in a restaurant setting, as well as their attitudes and knowledge regarding food allergies. Initially, the interviewers contacted 750 restaurant staff members in the designated divisional areas. Of these, 712 individuals voluntarily consented to participate in the study. Nevertheless, the data cleaning procedure excluded incomplete responses, resulting in a final sample size of 702 participants for the subsequent data analysis. A diverse representation of roles within the restaurant industry was achieved by the demographic composition of the restaurant personnel, which included chefs, assistant chefs, waiters, cleaners, and managers.

### 2.3 Instrumentation

A structured questionnaire was meticulously constructed for the purpose of data collection. To ensure that the questionnaire was accessible to participants, three bilingual researchers who were proficient in both English and Bengali translated the pre‐study questionnaire from English to Bengali, the native language of the target population. A back‐translation process was implemented to guarantee the translation's accuracy and integrity. A pilot study involving 30 participants was carried out to assess the clarity, cultural appropriateness, and internal consistency of the Bengali‐translated questionnaire. Based on feedback, minor revisions were made to improve item clarity. The internal consistency of each KAP construct was measured using Cronbach's alpha from the pilot data with values of 0.798, 0.849, and 0.727, demonstrating acceptable to good reliability. This necessitated the translation of the Bengali version into English by independent researchers who were not aware of the original questionnaire. It takes approximately 15 min to complete the questionnaire. The questionnaire was divided into four sections consisting of socio‐demographic and health‐related factors, food safety knowledge, attitudes, and practices. Section I was used to collect data on participants' gender, age, religion, marital status, residence area, education level, professional role, and job experience. It also collected data on general job satisfaction, personal history of food allergies, family history of food sensitivities, participation in food allergy training, and interest in learning more about food allergies. Section II on food safety knowledge included 10 questions that tested the participants' knowledge about food allergies. Participants were given three options for each question (“yes,” “no,” and “do not know”) to avoid selecting the correct answer by chance. Each correct answer received 1 point, while incorrect or “do not know” responses received 0 points. Sections III and IV were created to assess the participants' attitudes and practices about food allergies. This component comprised 10 questions evaluated using a 5‐point Likert scale, ranging from 1 (strongly disagree/never) to 5 (strongly agree/always). Reverse coding was applied for negative statements to ensure consistency in the data.

### 2.4 Ethics Statement

The study protocol was reviewed and approved by the Institutional Ethical Committee of the Patuakhali Science and Technology University (approval no. PSTU/IEC/2023/65 (4)). Before each interview, participants were fully informed about the objectives, procedures, potential risks, and benefits of the study. Written informed consent was obtained from all participants before data collection commenced. To maintain the integrity of the study, strict measures were implemented to guarantee confidentiality and anonymity of all data collected throughout the study.

### 2.5 Statistical Analysis

The Statistical Package for Social Science (SPSS) version 27 was used to conduct descriptive analysis, normality assessments, reliability analysis, Pearson's correlation analysis, and exploratory factor analysis (EFA). Confirmatory factor analysis (CFA) was performed using Analysis of Moment Structures (AMOS) version 23.0. A 95% confidence level was used throughout the analysis.

## 3 Results and Discussion

### 3.1 Descriptive Statistics

This survey included 702 restaurant workers, with the majority being male (93.9%). The majority of workers fell into the 21–30 and 31–40 age categories, which is consistent with findings from prior surveys on restaurant workers in Bangladesh, where the majority of staff are males aged 20–40 (Hasan et al. 2022). Male staff members make the majority of food preparation decisions, reflecting the overall trend of male domination in the restaurant profession (Dhaka Tribune 2022). More than half of the participants had received primary or secondary education, with only 6.7% having graduated, underlining restaurant workers' low educational attainment. A major 96.2% of workers indicated no prior training on food allergies. Almost 90% of the participants had more than 1 year of experience working in restaurants. Job satisfaction was indicated by 59.4% of workers, with 64.5% having a personal history of food allergies. Waiters (38%), followed by chefs (16.5%), assistant chefs (16.1%), and managers (18.2%), were the most common professional roles among participants. All participants identified as religious, with Muslims accounting for the vast majority (88.9%) (Table 1).

**TABLE 1:** Sample characteristics (*n* = 702).

Variables	Category	Frequency	Percentage %
Gender	Female	43	6.1%
	Male	659	93.9%
Age category (mean age: 33.17 ± 9.35)	≤ 20	31	4.4%
	21–30	294	41.9%
	31–40	224	31.9%
	41–50	125	17.8%
	> 50	28	4.0%
Religion	Muslim	624	88.9%
	Hindu	78	11.1%
Marital status	Unmarried	209	29.8%
	Married	476	67.8%
	Widowed/divorced	17	2.4%
Residence area	Urban	545	77.6%
	Rural	157	22.4%
Education qualification	No formal education	115	16.4%
	Primary	177	25.2%
	Secondary	215	30.6%
	Higher secondary	148	21.1%
	Hons and above^a^	47	6.7%
Professional role	Chef	116	16.5%
	Chef assistant	113	16.1%
	Waiter	267	38.0%
	Cleaner	78	11.1%
	Manager	128	18.2%
Working experience	Less than 1 year	71	10.1%
	1–3 years	213	30.3%
	3–5 years	182	25.9%
	More than 5 years	236	33.6%
Overall job satisfaction	Very dissatisfied	8	1.1%
	Dissatisfied	69	9.8%
	Satisfied	417	59.4%
	Very satisfied	208	29.6%
History of food allergy	No	169	24.1%
	Yes	453	64.5%
	Don't know	80	11.4%
Family history of food allergies	No	93	13.2%
	Yes	546	77.8%
	Don't know	63	9.0%
Participation in any training on food allergies	No	675	96.2%
	Yes	27	3.8%
Desired to get further information on food allergens	No	130	18.5%
	Yes	572	81.5%

^a^ Honors degree and above.

### 3.2 Construct Reliability and Validity

EFA was applied to extract the valid items from food allergy KAP. Items exhibiting a factor loading exceeding 0.40 were deemed acceptable (Baser et al. 2017; Soon 2019). Thus, a total of 2 items were deleted from food allergy management practice. The Kaiser‐Meyer‐Olkin (KMO) measure of sampling value for food allergy KAP was 0.807, 0.927, and 0.868, respectively. These values fulfill the criteria of validity (> 0.60) (Hair Jr. et al. 1988). The reliability of all extracted items was assessed using Cronbach's alpha coefficients, which exceeded the threshold of 0.70. This result indicates an acceptable level of internal consistency among the items (Hair 2010).

To ensure that the extracted items aligned appropriately with the empirical data, a CFA was performed together with an EFA. This additional test sought to enhance the construct validity of the latent factors. In relation to convergent validity, the confirmation of latent factors was achieved through the evaluation of factor loadings (> 0.40) and composite reliability (CR) (> 0.70) as indicated by Soon (2019) and Hair (2010). The factor loadings for food safety KAP items, as presented in Tables 3, 4, 5, varied between 0.41 and 0.85, with CR values surpassing 0.70. Increased factor loadings suggest a stronger correspondence of items with the model. In addition, to establish convergent validity, the average variance extracted (AVE) must exceed the minimal threshold of 0.5. However, Fornell and Larcker (1981) argue that convergent validity is still satisfactory if CR exceeds 0.6, even if AVE is less than 0.5. In our analysis, the KAP components have corresponding AVE values of 0.328, 0.522, and 0.427. Although knowledge and practice's AVE was below the 0.5 cutoff, all components' CR scores were over 0.6, suggesting adequate convergent validity. These results validate the measurement model's internal validity. As a result, we kept the knowledge and practice constructs since their reliability was adequate to ensure convergent validity. Additionally, the measurement items were adapted from established, validated instruments and reviewed for their suitability within the context of food allergies.

### 3.3 Discriminant Validity

Table 2 shows the Fornell‐Larcker criterion, with the square root of the AVE represented along the diagonal. To ascertain discriminant validity, the diagonal values must exceed the off‐diagonal correlation values for each construct. The findings demonstrate that the square roots of the AVE for knowledge (0.573), attitude (0.722), and practice (0.653) exceed their respective inter‐construct correlations, hence affirming discriminant validity (Fornell and Larcker 1981).

**TABLE 2:** The Fornell‐Larcker criterion.

Variables	Knowledge	Attitude	Practice
Knowledge	0.573		
Attitude	0.171^a^	0.722	
Practice	0.135^a^	0.606^a^	0.653

*Note:* Diagonals represent the square root of the AVE.

^a^ Pearsons's correlation is significant at the 0.01 level (2‐tailed).

### 3.4 Descriptive Statistics of Measurement Items

The findings in Table 3 indicate that 87.7% of respondents acknowledged that even a small amount of allergenic food could trigger an allergic reaction. This aligns with earlier research indicating that restaurant and food service staff possess a significant awareness of the risks associated with food allergens (Lee and Xu 2015; Lessa et al. 2016; Loerbroks et al. 2019; McAdams et al. 2018; Radke et al. 2016; Soon 2019; Wham and Sharma 2014). However, other studies have reported a lower level of such knowledge among staff (Eren et al. 2021; Sogut et al. 2015; Soon 2018b). The higher percentage indicates a robust understanding among staff regarding the seriousness of food allergies and the dangers associated with even minimal allergen exposure. The observed discrepancies could potentially indicate variations in training methodologies, regional policy implementations, or educational resource availability. In our study, the majority of staff (91.2%) believe that food allergies and food intolerances are separate. This contrasts with Bujaka and Riekstina‐Dolge (2019), who found that many restaurant employees feel that food allergies and food intolerances are the same thing or are unaware of their differences. Furthermore, 75.8% of restaurant staff were aware that food allergies cannot be eliminated by heating, roasting, or deep‐frying at high temperatures, consistent with similar studies (Lee and Xu 2015; Loerbroks et al. 2019; McAdams et al. 2018; Soon 2019; Wham and Sharma 2014). In contrast, Eren et al. (2021) found that 43.8% of participants were unaware of this fact, indicating a knowledge gap regarding the fact that cooking methods do not eliminate food allergies. A large majority of respondents (88.9%) agreed that simply removing allergens, such as nuts, from a finished meal does not guarantee the safety of customers with food allergies. This high level of awareness is consistent with prior research, which has highlighted the significance of comprehensive allergy management strategies (Loerbroks et al. 2019; McAdams et al. 2018; Radke et al. 2016). In contrast, Nasseredine et al. (2021) and Lee and Xu (2015) found over 40% of participants expressing ambiguity or lack of awareness of the safety consequences of allergen elimination alone. Finally, 88.2% of staff members identified epinephrine as the most effective drug for treating severe allergic responses, consistent with research conducted among Canadian restaurant staff (McAdams et al. 2018). However, Lee and Xu (2015) observed that only 60% of participants answered this correctly, showing a lower level of knowledge than our results. This discrepancy may be influenced by differences in national food safety training policies, cultural emphasis on allergy awareness, or the frequency and quality of training programs available to staff. The majority of our participants demonstrated extensive knowledge of food allergies; the opposing findings from research in Poland and Turkey highlight the important need for improved education and training for restaurants. Implementing systematic educational interventions may greatly improve knowledge and preparedness, thereby protecting individuals with food allergies (Wojtyniak et al. 2013; Sogut et al. 2015).

**TABLE 3:** Frequencies, item loadings, and validities for reliability test and convergent validity of items in knowledge toward food allergy (*n* = 702).

Items	Questions (Cronbach's *α* = 0.848)	Frequency (%)^a^	Standard loadings	Composite reliability	Average variance extracted
K1	Food allergies can develop from small amounts of food	616 (87.7)	0.59	0.827	0.328
K2	For children, cow milk is a common allergen	586 (83.5)	0.52		
K3	Allergic responses may occur within a few hours following the consumption of food	619 (88.2)	0.71		
K4	Food allergies are similar with food intolerance^b^	640 (91.2)	0.64		
K5	Cross‐contact occurs when proteins from one food mix with another	626 (89.2)	0.67		
K6	Taking out an allergen from a finished meal, like nuts, might be all that's needed to make it safe for a customer with a food allergy^b^	624 (88.9)	0.56		
K7	Food allergies can be eliminated by heating, baking, or deep‐frying meals at high temperatures^b^	532 (75.8)	0.53		
K8	Separate equipment should be used for products containing allergens.	565 (80.5)	0.58		
K9	Allergic reactions to food might cause difficulty breathing	537 (76.5)	0.45		
K10	The most effective medication for managing a severe food allergy reaction is epinephrine	619 (88.2)	0.41		

^a^ Indicates correct response frequency and percentage.

^b^ Indicates questions are reverse‐coded. These items were reverse‐coded during analysis to ensure consistent scoring direction, where higher scores indicate greater knowledge accuracy.

The findings presented in Table 4 indicate a predominantly positive attitude among staff regarding food allergy management. The high mean scores suggest a strong acknowledgment of their responsibilities (A1, 3.72), the importance of allergen knowledge (A5, 3.88), a proactive approach in adapting recipes (A6, 4.07), and providing allergen‐safe alternatives (A9, 4.01). These results align with studies in Canada, staff supported customize dishes to accommodate customers' food allergy requests (McAdams et al. 2018; Radke et al. 2016). However, the reduced score for interest in training (A3, 3.41) and the concerns regarding emergency handling (A7, 3.76) reveal gaps in both confidence and preparedness. McAdams et al. (2018) observed that restaurant employees strongly agreed that the food allergy training and education should be mandatory for all food service staff. McAdams et al. also found uncertainty among restaurant staff regarding their ability to handle food allergy emergencies at work, aligning with the findings of our study. Staff members in our study may not fully understand the importance of food allergy training, as most of our participants had never received training on food allergies. The moderate score for A2 (3.71) indicates a potential underestimation among some staff regarding the importance of food allergies within the restaurant industry. Implementing targeted training and establishing clearer allergen policies may significantly enhance staff competence, thereby contributing to improved overall food safety practices.

**TABLE 4:** Mean scores, item loadings, and validities for reliability test and convergent validity of items in attitude toward food allergy (1: strongly disagree to 5: strongly agree) (*n* = 702).

Items	Questions (Cronbach's *α* = 0.911)	Mean ± SD^a^	Standard loadings	Composite reliability	Average variance extracted
A1	Managing food allergies is a crucial aspect of my responsibilities	3.72 ± 0.94	0.73	0.915	0.522
A2	I don't think food allergies are a significant problem for the restaurant business^b^	3.71 ± 0.78	0.66		
A3	I am interested in attending food allergy training courses/workshops to gain a deeper understanding of food allergies	3.41 ± 1.09	0.59		
A4	I trust that providing correct allergen information to customers will reduce food allergy reactions	3.77 ± 0.83	0.78		
A5	Having a thorough knowledge of allergies to certain foods will prove beneficial in the work environment	3.88 ± 0.75	0.85		
A6	I will modify recipes or suggest alternatives for customers with food allergies	4.07 ± 0.73	0.69		
A7	I don't believe I can effectively handle a food allergy emergency at work^b^	3.76 ± 0.85	0.83		
A8	Food labels should indicate the presence of allergens	3.93 ± 0.78	0.57		
A9	Customers with food allergies should be given options based on their requirements	4.01 ± 0.71	0.68		
A10	Staff should be capable of figuring out menu ingredients when asked by a customer	3.89 ± 0.76	0.79		

^a^ Mean value was calculated by sum of total responses in each question divided by the total number of respondents.

^b^ Indicates questions are reverse‐coded.

Table 5 highlights the differences in food allergy management practices among participants. There is strong adherence to fundamental hygiene practices, including handwashing (P1, 4.44) and maintaining uniform cleanliness (P2, 4.25). Many studies have also reported similar findings to ours, suggesting that the majority of food handlers thoroughly wash their hands with soap and water and wear a fresh pair of gloves before preparing allergen‐free meals (Eren et al. 2021; Jianu and Goleţ 2019; Soon 2018a). This finding is important because proper handwashing and the use of new gloves play a crucial role in preventing cross‐contamination, particularly in the preparation of allergen‐free meals. We also found that restaurant staff cleaned and sanitized utensils to prevent cross‐contamination before preparing meals, which aligns with findings from other similar studies on this topic (Eren et al. 2021; Jianu and Goleţ 2019; Soon 2018a). In contrast, there is a marked lower adherence to essential precautions, such as verifying ingredients (P5: 2.83 ± 1.31) and remaking food when required (P8, 2.83). This contrasts with other studies that report positive actions taken in such scenarios (Eren et al. 2021; Soon 2018a). High‐pressure environments, especially during busy hours, might discourage staff from taking the time to remake meals, leading them to prioritize speed over safety. Practices that exhibit lower mean scores, such as double‐checking dishes (P6: 2.90 ± 1.30), underscore potential risk areas for allergic reactions. Shafie and Azman (2015) found that 48.7% of food handlers did not double‐check whether the food was safe from allergens when the meal was ready, which supports similar practices observed among restaurant staff in our study. Food handlers might not fully understand the processes for verifying that food is free from allergens, resulting in inconsistent practices, underscoring the need for targeted training and reinforced protocols for food safety.

**TABLE 5:** Mean scores, item loadings, and validities for reliability test and convergent validity of items in food allergy management practices (1: never to 5: always) (*n* = 702).

Items	Questions (Cronbach's *α* = 0.837)	Mean ± SD^a^	Standard loadings	Composite reliability	Average variance extracted
P1	I wash my hands and change gloves before making allergen‐free meals	4.44 ± 0.10	0.51	0.854	0.427
P2	I wear clean uniforms while preparing food	4.25 ± 1.17	0.59		
P3	I use the same tools for handling both allergenic and non‐allergenic foods^b^	3.13 ± 1.65	0.51		
P4	I take extra precautions when preparing the eight most prevalent food allergens	2.87 ± 1.36	0.76		
P5	I verify ingredients and read contents before using packaged foods	2.83 ± 1.31	0.79		
P6	If a customer has a food allergy, I double‐check to ensure the dish is allergen‐free	2.90 ± 1.30	0.67		
P7	I keep foods that contain allergens and foods devoid of allergens in the same places^b^	3.44 ± 1.25	< 0.40		
P8	If something goes wrong, I remake the food when serving a customer with a food allergy	2.83 ± 1.34	0.66		
P9	Before cooking, I ensure utensils are clean and sanitized to prevent cross‐contact	4.13 ± 0.96	< 0.40		
P10	I make an effort to attentively respond to customer inquiries about food allergies	4.21 ± 0.87	0.68		

^a^ Mean value was conducted by the sum of total response in each question divided by the total number of respondents.

^b^ Indicates questions are reverse‐coded.

### 3.5 Structural Model

The evaluation of the structural model in SEM was conducted using fit indices and variance‐explained estimates, as presented in Table 6. The *χ* ^2^/df ratio of 2.892 fell below the threshold of 3, while the RMSEA value of 0.052 remained within the acceptable range of less than 0.08, suggesting a favorable fit (Kline 2015; Hu and Bentler 1999; Browon and Cudeck 1993). The incremental fit indices—CFI (0.941), NFI (0.912), and TLI (0.930)—all surpassed the threshold of 0.90, thereby reinforcing the adequacy of the model (Hu and Bentler 1999; Kline 2015). The GFI value of 0.912 satisfied the criterion of being greater than 0.90, whereas the AGFI value of 0.889 fell just short but remains acceptable considering the complexity of the model (Hu and Bentler 1999; Kline 2015). Residual‐based measures, SRMR (0.075) and RMR (0.038), fell below the threshold of 0.08, suggesting that discrepancies are minimal (Hu and Bentler 1999). The parsimony indices, PCFI (0.799) and PNFI (0.775), surpassed the > 0.50 threshold, indicating a balance between fit and simplicity (Mulaik et al. 1989; Kline 2015).

**TABLE 6:** Goodness of fit indices for structural equation modeling of food allergy knowledge, attitude, and practice.

Fit indices	Model value	Accepted value	References
**Absolute fit measures**			
*χ* ^2^	928.350		
df	321		
*χ* ^2^/df	2.892	< 3	Kline (2015) and Schumacker and Lomax (2016)
Goodness of fit index (GFI)	0.912	> 0.90	Hu and Bentler (1999)
Adjusted Goodness of fit index (AGFI)	0.889	> 0.90	Kline (2015) and Hu and Bentler 1999
Root Mean square error of approximation (RMESA)	0.052	< 0.08	Hu and Bentler (1999) and Browon and Cudeck (1993)
Root Mean square residual (RMR)	0.038	< 0.08	Hu and Bentler (1999)
Standardized root mean squared residual (SRMR)	0.075	< 0.08	Hu and Bentler (1999)
**Incremental fit measures**			
Comparative fit index (CFI)	0.941	> 0.90	Hu and Bentler (1999) and Kline (2015)
Normed fit index (NFI)	0.912	> 0.90	Hu and Bentler (1999) and Kline (2015)
Tucker‐Lewis index (TLI)	0.930	> 0.90	Hu and Bentler (1999) and Kline (2015)
**Parsimony fit measures**			
Parsimony comparative fit index (PCFI)	0.799	> 0.50	Mulaik et al. (1989) and Kline (2015)
Parsimony normed fit index (PNFI)	0.775	> 0.50	Mulaik et al. (1989) and Kline (2015)

**FIGURE 2:**
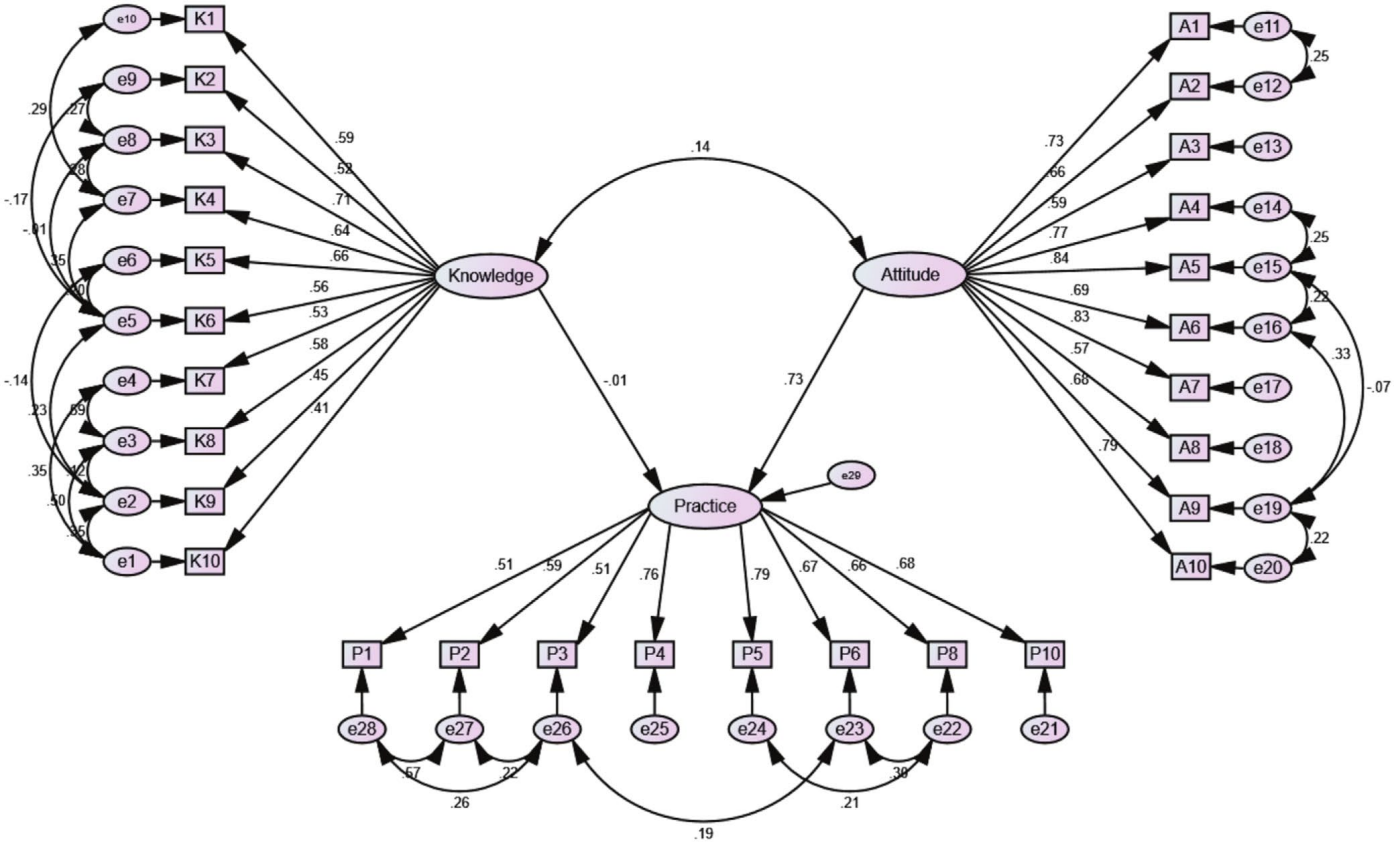
Structural equation modeling of food allergy knowledge, attitude, and practices among restaurant's staff in Bangladesh.

The findings demonstrated in Table 7 and Figure 2 highlight the standardized path coefficients, which indicate both the strength and direction of the direct relationships among the constructs. The initial hypothesis posited that knowledge of food allergies does not exert a direct influence on the practices related to food allergy management (H1). The results reveal a path coefficient of −0.012 for the relationship between knowledge and practice, accompanied by a critical ratio of −0.339 and a *p*‐value of 0.735. This suggests that the relationship does not reach statistical significance. The small negative coefficient indicates a minimal inverse relationship, supporting the hypothesis that knowledge alone may not directly influence management practices. Similar findings from earlier studies (Baser et al. 2017; Soon 2018b, 2019; Agwa 2023) corroborate our findings, showing that improved management practices are not always correlated with employees' knowledge of food allergies. However, according to a Costa Rican study, better management practices are associated with a higher level of knowledge of food allergies (López‐Calvo et al. 2022). Lee and Xu (2015) indicated that while training positively influenced knowledge, this knowledge alone proved inadequate for altering food safety practices. Research conducted on trained food handlers alongside studies of inadequately trained food handlers (Garayoa et al. 2011) indicates a disconnection between knowledge and the implementation of effective practices. The discrepancy between theoretical comprehension and practical application is a substantial barrier to the practical application of knowledge. For example, Saleh‐Langenberg et al. (2017) discovered that patients' knowledge of the proper use of epinephrine auto‐injectors did not always translate to their correct application in emergency situations (Saleh‐Langenberg et al. 2017). Furthermore, the training that restaurant personnel receive frequently is insufficient in terms of its practical application and profundity. Tan et al. (2023) demonstrated that restaurant managers and servers possessed varying degrees of food allergy knowledge, but there were significant variations in their perceived training requirements and food safety practices. In addition, the environment in which restaurant staff operate can either facilitate or impede the implementation of secure food handling practices (Ajala et al. 2010). For instance, employees may experience undue pressure to disregard appropriate allergen management practices, regardless of their level of expertise, if a restaurant prioritizes efficiency and speed over safety (Peiris et al. 2015). Therefore, for the conversion of knowledge into effective food allergy management practices, a multifaceted approach that integrates education, hands‐on training, and a supportive work environment is needed.

**TABLE 7:** Estimates of hypothesis paths for food allergy knowledge, attitude, and practice.

Hypothesis	Paths	Estimates	C.R.	*p*
H1	Knowledge → Practice	−0.012	−0.339	0.735
H2	Attitude → Practice	0.733	14.018	0.000
H3	Knowledge ↔ Attitude	0.136	2.889	0.004

The results indicate a positive and significant relationship between staff members' attitudes toward food allergies and their management practices (*β* = 0.733, *p* < 0.05), supporting Hypothesis 2 (H2). Figure 2 suggests that with each unit increase in staff members' attitudes, good management practices improve by 0.733 units. A positive attitude among restaurant staff demonstrates their dedication and motivation to manage food allergies effectively, ensuring customer protection from potential allergic reactions. Our findings on this issue are consistent with those of other studies conducted on similar topics (Baser et al. 2017; Soon 2018b; López‐Calvo et al. 2022). López‐Calvo et al. also reported that improved attitudes toward food allergens are linked to improved management practices, which aligns with our findings (López‐Calvo et al. 2022). Staff members who maintain positive attitudes are generally more aware of the risks related to food allergens, resulting in more careful and attentive management practices. In contrast, knowledge alone showed no significant effect (*β* = −0.012), possibly because theoretical understanding does not automatically translate to practical understanding in busy restaurant environments where time constraint, workflow pressures, and limited managerial support may override knowledge. However, a similar study conducted on UK consumers presents a contrasting perspective, revealing that high scores on attitude items did not consistently result in effective management practices, and there was no significant correlation between attitudes and management practices regarding food allergens (Soon 2019). This discrepancy may be the result of differences in the operational contexts of restaurants, as well as varying levels of awareness and education among staff and consumers. A large percentage of participants in our study disclosed that they had a personal history of food allergies. This likely increased their awareness of the severity of allergic reactions and contributed to their positive attitudes toward managing food allergies. These results emphasize that although knowledge is necessary, it is insufficient alone; effective food allergy management requires cultivating positive staff attitudes through empathy‐focused training and addressing operational challenges that obstruct the application of safety protocols.

Our study found a positive and significant correlation between food allergy knowledge and attitude (*β* = 0.136, *p* < 0.05), supporting Hypothesis (H3). This indicates that knowledge and attitude together have a dependent influence on practices. An increase in staff members' knowledge about food allergies will likely lead to an improvement in their attitudes toward managing food allergies effectively. Our results are consistent with the conclusions of previous studies that were conducted in comparable contexts (Agwa 2023; Baser et al. 2017; López‐Calvo et al. 2022; Soon 2019). The findings underscore the importance of enhancing knowledge among food service staff, which appears to result in more responsible and careful management of food allergens, thereby promoting safer dining experiences for individuals with allergies. However, another study found an insignificant relationship between food allergen knowledge and attitude among Malaysian consumers (Soon 2018b). The differences in findings may come from variations in cultural perspectives, levels of awareness, or the effectiveness of educational outreach related to food allergies. Furthermore, the observed discrepancies between our study and Soon's findings in Malaysia may be attributed to methodological differences, particularly in the nature and phrasing of the knowledge and attitude assessment questions.

### 3.6 Practical Implications of the Study

According to the findings, practical implications for improving food allergy management in restaurants include implementing integrated training programs that not only improve knowledge but also focus on building good staff attitudes, as these attitudes serve as a vital link to better practices. Specifically, training modules should include allergen identification, cross‐contact prevention, effective customer communication, and emergency response protocols. These should be delivered through mandatory annual workshops, supported by periodic refresher sessions. Successful initiatives such as Canada's Food Allergy Canada training program can serve as a model for developing context‐specific training in Bangladesh (Food Allergy Canada 2023). Hands‐on training, refresher courses, and visual aids are critical for closing knowledge gaps and improving emergency readiness. To reduce cross‐contact risks, structural improvements such as specialized allergen‐free preparation facilities and effective ingredient verification methods are required, along with explicit rules for reconstructing meals as necessary. Transparent menu labeling and open communication with customers are essential for safeguarding those with food allergies. Additionally, collaboration with the Bangladesh Food Safety Authority is recommended to standardize allergen management protocols across the restaurant industry, ensuring nationwide compliance and accountability. Leadership must aggressively foster a culture of responsibility and accountability by ensuring that employees prioritize safety over speed, particularly during peak hours. Continuous monitoring, including audits, feedback mechanisms, and coordination with health authorities, will ensure that best practices are followed and encourage continuous development. These steps not only make dining areas safer, but they also increase consumer trust and confidence, which improves the restaurant's reputation and customer happiness.

### 3.7 Limitations

The current study had a few limitations. For example, using convenient sampling procedures for selecting respondents reduces the generalizability of the results. The recruitment of restaurant employees who have voluntarily decided to participate may cause selection bias. Individuals with a stronger interest or understanding of food allergies may have been more likely to participate, skewing the data toward more positive KAP outcomes. The vast majority of participants stated that they had no prior food allergy training. This lack of training may reflect a larger industry gap, but it may also limit the depth of practical ideas given by respondents. Because participants had limited exposure to formal training, it is challenging to evaluate the potential impact of such training on food allergy KAP. Future research should address this gap by including participants with a broader range of training experiences. The sample is highly skewed toward male participants, with some female participants. This gender gap may restrict the findings' generalizability since male and female restaurant employees may have differing roles, responsibilities, and levels of exposure to food safety training, all of which could influence their KAP about food allergies. Future research should aim for more balanced gender representation to better reflect the perspectives and practices of all employees. Given the face‐to‐face data collection, social desirability bias may have caused participants to overstate their KAP related to food allergies. This could lead to inflated knowledge levels or a more favorable attitude and practices than are actually present in the broader restaurant workforce, affecting the accuracy of the findings and should be considered while interpreting the results. The cross‐sectional nature of the sample prevents the establishment of cause‐effect relationships. The KAP model employed in this study was not validated using independent datasets. Although the observational study may serve as indirect validation, the lack of direct validation may raise concerns about the model's resilience and adaptability to other restaurant environments. The study does not fully account for how Bangladesh's cultural, economic, and regulatory context may uniquely shape restaurant staff's KAP, potentially limiting the generalizability of the findings. Future research should therefore incorporate comparative analysis, cross‐country validation, and longitudinal design to assess the stability and predictive validity of the SEM while accounting for local and regional variations.

## 4 Conclusion

Restaurant staff exhibited a strong awareness of food allergies and employed effective strategies to prevent cross‐contamination, thereby safeguarding customers with allergies. However, their food allergy management practices were found to be insufficient when it came to double‐checking or rectifying mistakes related to the preparation of food. Additionally, staff members conveyed a lack of confidence regarding their participation in food allergy training courses or workshops aimed at enhancing their knowledge of food allergies. Many of the staff members did not agree on whether managing food allergies was part of their responsibilities. The SEM indicated that the knowledge, attitudes, and practices of the restaurant staff regarding food allergens exhibited a satisfactory fit. While knowledge of food allergies does not directly influence management practices, attitudes toward food allergies do have a significant impact on those practices. A substantial correlation was discovered between knowledge and attitude as well. Addressing gaps in training and clarifying responsibilities related to food allergies are essential steps toward improving staff confidence and competence. By fostering a culture of safety and accountability, restaurants can ensure a safer dining experience for all customers, particularly those with food allergies, ultimately contributing to greater customer satisfaction and trust.

## Author Contributions

**Nitai Roy:** conceptualization, methodology and study design, acquisition of data, supervision, validation, visualization, formal analysis and interpreting data, writing – original draft, writing – review and editing. **Sultan Mahmud Imran:** data curation, formal analysis and interpreting data, visualization, and writing – review and editing. **Sourav Chandra Debnath:** data curation, visualization, writing – review and editing. **Abdullah Al Adib:** visualization, writing – original draft, writing – review and editing. **Samia Sultana:** visualization, writing – review and editing. **Sumana Mahmud:** visualization, writing – review and editing. **Rejwana Rashid:** visualization, writing – review and editing. **Ekhtear Hossain:** visualization, writing – review and editing. **Farhadul Islam:** visualization, writing – review and editing. **Kamal Krishna Biswas:** visualization, writing – review and editing. All the authors approved the final version of the manuscript.

## Conflicts of Interest

The authors declare no conflicts of interest.

## Data Availability

Data will be made available on request.
